# Women Have Tendons… and Tendinopathy: Gender Bias is a “Gender Void” in Sports Medicine with a Lack of Women Data on Patellar Tendinopathy—A Systematic Review

**DOI:** 10.1186/s40798-022-00455-6

**Published:** 2022-06-07

**Authors:** Camilla Mondini Trissino da Lodi, Maria Paola Landini, Emanuela Asunis, Giuseppe Filardo

**Affiliations:** 1 Service of Orthopaedics and Traumatology, Department of Surgery, EOC, 6900 Lugano, Switzerland; 2 Scientific Direction, Istituto Ortopedico Rizzoli, 40136 Bologna, Italy; 3 II Clinica, IRCCS Istituto Ortopedico Rizzoli, 40136 Bologna, Italy; 4 Applied and Translational Research Center, IRCCS Istituto Ortopedico Rizzoli, 40136 Bologna, Italy; 5 Facoltà Di Scienze Biomediche, Università Della Svizzera Italiana, Via Buffi 13, 6900 Lugano, Switzerland

**Keywords:** Patellar tendinopathy, Gender bias, Sports medicine, Tendinopathy

## Abstract

**Introduction:**

Patellar tendinopathy is one of the most common musculoskeletal problems associated with sport. While commonly perceived as a predominantly male problem, recent epidemiological studies revealed that it also affects a large number of sport-active women. The aim of this systematic review was to understand how the available treatments apply to women affected by patellar tendinopathy.

**Methods:**

We analysed the available literature with a systematic review on three databases (PubMed, Cochrane, Web of Science) on February 2021, retrieving a total of 136 studies published from 1983.

**Results:**

The overall scientific field offers an astonishingly low number of data on treatment results referring to only 78 women (2%) in the entire literature. Only 5% of the retrieved articles considered focusing only or mostly on men to be a limitation.

**Conclusions:**

Women represent only a minority of patients studied for this topic. The few documented cases are further fragmented by being related to different treatments, thus basically offering no solid evidence for results and limitations of any therapeutic approach in women. This literature analysis showed a greater gender gap than what is recognized in science and general medicine; it showed a gender blindness in sports medicine when investigating a common problem like patellar tendinopathy.

## Key Points

- Patellar tendinopathy is a common pathology with a high prevalence among athletes, for which several conservative therapies have been developed.
- Women are at great risk of sustaining tendon and ligament injuries during physical activities due to several factors including hormonal fluctuation.
- Analysis of sex-specific data is a simple yet critical step towards gender equality, which is too often omitted despite the many indications provided by different authorities.
- There are no data available in the current literature to support the potential and limitations of the available treatments of patellar tendinopathy in women.

## Introduction

Awareness of the need for gender equality is growing in every field, including sports medicine. In fact, sex and gender disparity encompasses many aspects of our societies, including science and medical treatments. Regarding sex, current literature refers to the biological and physiological features characterizing male and female individuals, while gender represents socially constructed roles, behaviours and identities of female, male, and gender-diverse people [1, 2]. In this context, gender bias refers to the lack of scientific data concerning half of the human population, due to the fact that for cultural reasons women are often not taken into account [3].

The gender data gap is in this context a consequence of the tendency to consider males (Caucasian males, usually) the norm or the standard and females as deviant [4]. In 1986, the NIH (National Institute of Health) encouraged increased female inclusion in clinical research, calling for justification in cases of exclusion [5, 6]. This resulted in the 1993 Revitalization Act that required female participation in NIH-funded clinical research [7]. In 1997, the FDA (Food and Drug Administration) upheld the analysis of clinical data by sex [8] and in 2001 a report affirming that sex is an important variable that must be considered in all aspects and at all levels of biomedical research was published by the Institute of Medicine [9]. Between 2005 and 2008, the GenderBasic project in Europe supported scientific studies focusing on sex and gender and the importance of distinguishing outcomes by sex [10]. A few years later, the European Association of Science Editors (EASE) established a Gender Policy Committee with the aim to develop a set of guidelines for reporting of Sex and Gender Equity in Research (SAGER) [2]. During the same period, the Canadian Institute of Health mandated a justification for any article if only one sex was considered [11], and in 2014 the NIH started promoting the use of both sexes also for preclinical animal research [12].

Sports medicine research should also identify factors that may affect risk or prognosis, including patient sex. Up to the 1970s, the suitability of sports competition for girls and women was still being questioned in some circles, exemplified by publications titled “Women in Sports: Some Misconceptions” [13] and “Inferiority of Female Athletes: Myth or Reality?” [14]. Some of the myths surrounding female athletic participation have been challenged, and now there are studies that do examine dissimilarities between the sexes focusing on differences in injury risk or prognosis of conditions such as concussions and ACL injuries [15]. The aforementioned gender equity indications have also been proposed with the aim to decrease female underrepresentation and underline the importance of sex-disaggregated data in order to provide women “benefits of care based on good, applicable evidence” [16].

Patellar tendinopathy is a common musculoskeletal problem associated with sport [17–19]. Often referred to as “jumper’s knee”, it results from overuse in activities that require jumping, running, squatting, or rapid changes in direction. This disorder affects athletes in many sports. The prevalence of jumper's knee has been estimated to be up to one-third among elite basketball players and between 40 and 50% among high-level volleyball players [18], and there is a high prevalence among soccer players, sprinters, and jumpers as well [20]. Prominent features include histopathological changes such as disorganization of collagen fibres, an increase in the number of vessels and sensory nerves, an increase in hydrated components of the extracellular matrix and [21], ultimately, apoptotic death of tenocytes leading to an overall breakdown of tissue organization [22]. Characterized by microscopic ruptures with degenerative changes in the patellar tendon, most frequently in its origin on the inferior pole of the patella, this condition results in substantial pain and reduced performance in sports. Most patients report persistent pain and disability, with some experiencing long-term limitations. There is evidence showing that the average duration of pain and reduced function can be nearly 3 years, and that at 15 years follow-up 53% of patients reported having to quit their sports career because of their knee problem [23]. Thus, patellar tendinopathy can severely affect patients even to the point of abandoning their athletic career. Moreover, it is also common as a work-related condition and even in the sedentary population causes pain and dysfunction that impair activities of daily living [24]. While commonly perceived as a predominantly male problem, recent epidemiological studies revealed that it also affects a large number of sport-active women [20, 25–31].

The purpose of this article was to systematically review the literature to determine if the available treatments have been studied considering gender-related differences and thus apply to women affected by patellar tendinopathy.

## Methods

### Literature Research

**Fig. 1:**
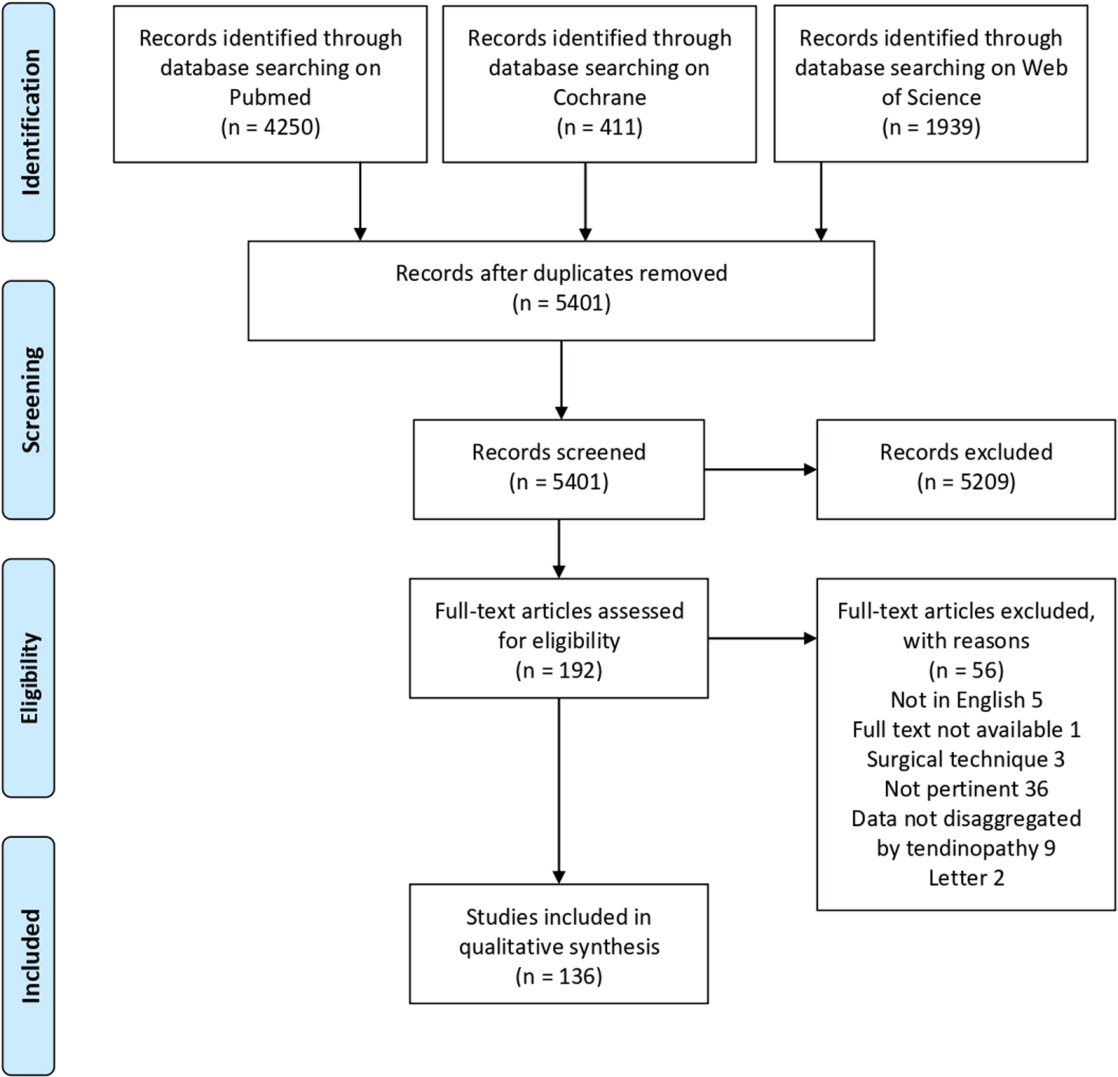
PRISMA (Preferred Reporting Item for Systematic Meta-Analyses) flow chart of the article selection process

A systematic review of the literature was performed on February 1, 2021, using three bibliographic databases: PubMed, Web of Science, and Wiley Cochrane Library. The following search terms were used for initial screening “((patellar) AND ((tendinopathy) OR (tendinitis) OR (tendon pathology))) AND (treatment))”. No limitations based on the publication time were made. The guidelines for Preferred Reporting Items for Systematic Reviews and Meta-analysis (PRISMA) were used. A flow chart of the articles’ selection for the qualitative data synthesis is reported in Fig. 1.

### Articles Selection and Data Extraction

Two authors (CMTDL, EA) performed the article selection independently, and duplicate articles were removed. Articles were included if they met the following criteria: clinical reports of any level of evidence, written in English language, with no time limitation, on therapies used to treat patients affected by patellar tendinopathy. Articles written in other languages, animal studies, and reviews were excluded. After screening titles and abstract, full text for the articles that met inclusion criteria were reviewed. In case of disagreement between the two reviewers, a third author was consulted (MPL).

An electronic table for data extraction was created prior to the article using Excel (Microsoft). The relevant data were then extracted: title, first author, year of publication, journal, type of article, level of evidence, population characteristics, type of treatment, article limitations, outcomes, and outcomes disaggregated by sex.

The included articles were further divided into three different groups based on the treatment: conservative treatment, injectable treatment, and surgical treatment. If an article compared two distinct approaches, they were counted individually.

### Statistical Analysis

Absolute numbers and percentages were used to report the number of articles and the sex of patients enrolled.

For each article, sex matching was calculated as the ratio of the two sexes, multiplied by 100, using the lesser number of subjects (male or female) in an individual article as the numerator and the greater number of subjects (male or female) as the denominator. For instance, an article with 50 women and 50 men would result in a 100% sex matching, as 50/50 × 100 = 100% [32].

### Institutional Board Review and Funding Source

IRB approval was not required because all data were extracted from previously published studies. No external funding was received for the initiation or completion of this article.

## Results

### Article Selection

The database search identified 6600 records, of which 1199 were identified as duplicates and removed. Of the remaining 5401 records, 192 full-text articles considered suitable for inclusion were assessed for eligibility. Fifty-six articles did not meet the inclusion criteria and were excluded, leading to a total of 136 studies used for the analysis. The articles were all published between 1983 and 2020 with most of the studies being published in the last two decades (116/136 articles, 85%).

### Clinical Articles

#### Article Characteristics

Of the 136 clinical articles, six (4%) did not specify the sex of the enrolled patients. The remaining 130 articles (96%) stated the sex of the patients, two (1%) of which included only women, 25 (19%) articles included only men and 103 (80%) included both sexes. Of the 103 articles including both sexes, only one separated data by sex [33] and two referred to a general outcome for women, but without specifying their data [34, 35]. Seven other articles did perform sex-specific analysis, but without specifically discussing their data [36–42].

Only six (5%) articles considered enrolling only or mostly men as a limitation [43–48].

#### Representation of Women

Overall, 4142 patients were studied. Besides the 6% of patients for whom sex was not specified, the remaining 3894 patients were represented by 78% men (3022), while women accounted for 22% (872) of the patients. This percentage did not increase when evaluating only the high-level studies: the 41 randomized controlled trials enrolled a total of 310 women (22%) and 1121 men (78%).

**Fig. 2:**
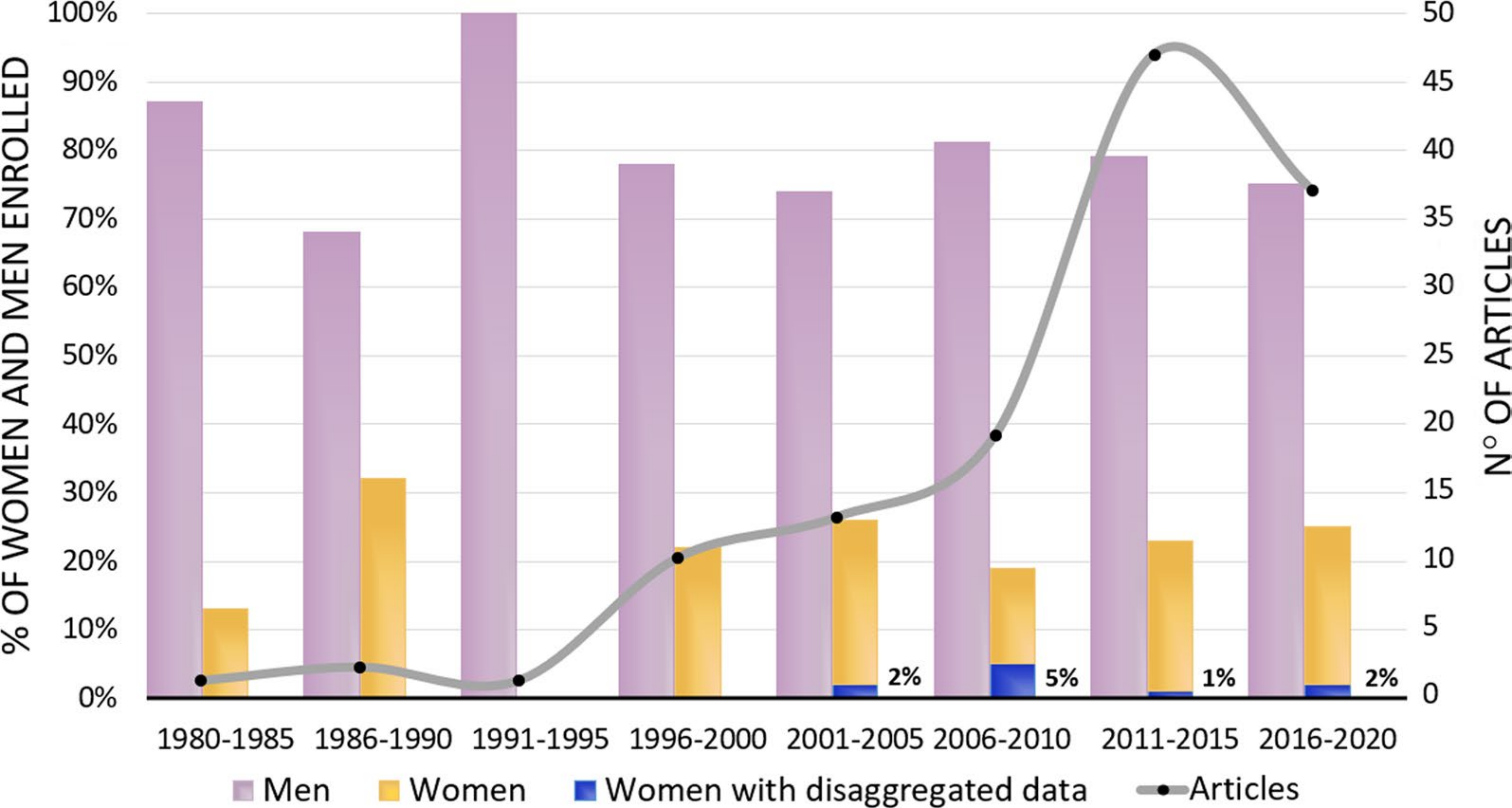
Percentage of women and men enrolled in patellar tendinopathy related research retrieved in this systematic review, and corresponding number of articles published from 1980 to 2020

Analysis of the available data on the treatment of patellar tendinopathy was specified for 78 women, which represents 9% of all the enrolled women, and 2% of all patients studied in the literature on this topic. While the number of articles increased over the years, the percentage of female inclusion did not, since the highest value (32%) was reached between 1985 and 1990. In the last five years, between 2016 and 2020, 37 articles on the treatment of patellar tendinopathy were published, including 25% women (Fig. 2).

Women were represented more in conservative treatments (50%), while they were less present in the injectable (28%) and surgical treatments (22%). The results of conservative, injectable, and surgical treatment in women were reported in 50, 7, and 21 cases, respectively.

#### Sex-matching

Of the 103 studies enrolling both sexes, 11% had less than 10% women, 42% had less than 20% women, 16% had less than 30% women, and 23% had less than 50% women. Only 8 articles (8%) included more than 50% women.

Sex-matched analysis demonstrated that only 17/103 studies (16%) matched the sex of more than 50%, only 6/103 studies (6%) had a sex matching of more than 80%, and only 2/103 studies (1%) had a sex-matching of 100%, enrolling the same number of women and men.

## Discussion

The main finding of this systematic review is that there is a large gender gap in articles focused on the treatment and outcome of patellar tendinopathy in women.

A plethora of therapeutic strategies have been developed to address patellar tendinopathy, including physical therapy, and other conservative approaches independently or concurrently applied to favour healing of this challenging pathological condition, such as electrotherapy, massage, taping, anti-inflammatory medication, extra-corporeal shock-wave therapy, and different types of injections (platelet-rich plasma, autologous plasma, cells derived from dermal fibroblasts, bone marrow-derived stem cells). In cases where conservative treatment fails, surgery may be considered [49].

To understand how these treatments apply to women affected by patellar tendinopathy, we analysed the available literature, looking at female representation and if data disaggregation allows for a proper understanding of indications and results of these treatments in women. This systematic review of the literature was performed to retrieve data related to women by including all studies focusing only on women and those separating data by sex: only 78 women (from 10 studies) in the entire literature. This represents less than 2% of all patients studied for this topic. These cases are further fragmented by being related to different treatments, thus basically offering no solid evidence for results and limitations of any therapeutic approach in women. Even though evaluating the treatment of patellar tendinopathy has increased over the past several years, the attention towards proper gender representation has not (Fig. 2). Even worse, this has not even been perceived as an issue, as only 5% of the retrieved articles considered focusing only or mostly on men to be a limitation. Sport participation of women has increased substantially in the last few decades and therefore the number of women affected by patellar tendinopathy has also increased [50]. Women are at greater risk of sustaining tendon and ligament injuries during physical activities [17, 51–54], which has been partially explained by the potential effect of hormones, since it has been demonstrated that both ligaments [55] and tendons contain oestrogen receptors [56]. Increased concentration of oestrogen can lead to the inhibition of collagen synthesis in women [57, 58], as confirmed by the lower patellar tendon collagen synthesis rate compared to men, both at rest and after exercise [59]. In the patellar tendon of women, a higher mRNA expression of collagen type III was found. This is considered a marker of injured tissue implying smaller and less organized fibrils which could easily lead to damage and rupture [60]. In addition, lower tendon stiffness has been reported in women [61], leading to a worse adaptation to mechanical loading and a reduced tendon hypertrophy response after habitual training [62, 63]. The concept that sex hormones have a role in relation to injury is further supported by research showing a lower tendon collagen synthesis in patients taking oral contraceptives [64], while in post-menopausal women tendon stiffness matches that of men [65]. These sex-related differences can affect the likelihood of developing a tendon injury, as well as the response to available treatments, in terms of success rates and adverse effects. Additionally, hormones can affect pain perception, which further complicates the assessment of tendinopathy and treatment results, and further research is necessary to better understand the impact of patient sex on tendon, treatment options, and outcomes [66–69].

Differences in pharmacokinetics between men and women can make women more prone to experience adverse drug reactions, as evidenced and exemplified by the Zolpidem case [70]. This drug was approved and commercialized with the same dosage for both sexes. Ten years later, researchers pointed out that levels in women were high enough to cause next-morning impairment lowering their performance in tasks that require alertness, including driving, and therefore the FDA started recommending women take half the dose [71, 72]. Besides the side effects, male overrepresentation can lead to the production of drugs with less or no effect at all in women [73]. Women and men can differ in terms of outcomes, re-admission rates, and post-operative complications [74, 75]. In this light, reporting data stratified by sex is necessary to understand the risks of the different treatment options. However, it is not enough to enroll women within clinical studies to overcome the risk of gender inequality. For example, in the orthopaedic field Kalliainen et al. analysed hand surgery research: 47% of the enrolled patients were women but still, only 23% of the studies calculated statistics by sex [76]. Even though a 19% to 30% increase in sex-specific analyses has been reported between 2000 and 2010 in five high-impact orthopaedic journals [8], another analysis of the surgical literature found that only 38% reported data by sex, 33% analysed data by sex, and 23% included a discussion of sex-based results [32]. Moreover, only 2% of these articles matched the sex of included subjects by 100%, having the same number of enrolled men and women, and only 18% of the studies were able to reach an 80% match between male and female patients.

There are limitations to this review. First, this analysis is focused only on one pathology in the orthopaedic field, and thus it cannot be assumed that these findings are consistent for all orthopaedic research. Second, the data we extracted are limited to the phenotypic concept of sex with no regard to the gender issue, since no mention of it can be found in the articles analysed. Also, it should not be forgotten that a binary representation of sex/gender can lead to the exclusion of transgender, gender-fluid, and intersex individuals and that a simple disaggregation by sex could mask other subtleties, such as socioeconomic status, ability, and ethnic background, thus not adequately addressing the needs of different groups of people.

From 1983 to the present day, the number of women enrolled in studies on patellar tendinopathy has not increased, and even though most studies included both sexes, there is a persisting lack of awareness of the importance of disaggregating data by sex. This is not to say that an equal number of men and women must be enrolled in every study. It might not always be feasible, but it is important to provide sufficient data granularity recording the sex-specific outcomes to allow the data to be abstracted for use in a subsequent meta-analysis. In this way, it will be possible to understand if different treatments need to be considered for women and men. Disaggregating data is a simple yet critical step towards gender equality, which is too often omitted despite the many indications provided by different authorities. Research progresses, but gender bias persists, and we keep addressing only half the population. Gender bias persists in orthopaedics and sports medicine and can no longer be ignored. More women should be enrolled in scientific studies and sex-based analysis should be implemented systematically to guarantee women a high level of care and better outcomes in the management of patellar tendinopathy*.* We have the duty to offer women appropriate treatments that are no longer based on male data.

## Conclusions

Women represent only a minority of patients studied for patellar tendinopathy. The few documented cases are further fragmented by being related to different treatments, thus basically offering no solid evidence for results and limitations of any therapeutic approach in women. This literature analysis showed a greater gender gap than what is recognized in science and general medicine; it showed a gender blindness in sports medicine when investigating a common problem like patellar tendinopathy.

## Data Availability

The datasets used and/or analysed during the current systematic review are available from the corresponding author on reasonable request.
